# Morphological and Pomological Characterizations of Peach [ *Prunus persica* (L.) Batsch] Accessions

**DOI:** 10.1002/fsn3.70501

**Published:** 2025-06-27

**Authors:** Ali Khadivi, Farhad Mirheidari, Abdolvahid Saeidifar, Younes Moradi, Yazgan Tunç, Daya Shankar Mishra

**Affiliations:** ^1^ Department of Horticultural Sciences, Faculty of Agriculture and Natural Resources Arak University Arak Iran; ^2^ Ministry of Agriculture Jihad Zahedan Iran; ^3^ Republic of Türkiye, Ministry of Agriculture and Forestry, General Directorate of Agricultural Research and Policies Hatay Olive Research Institute Directorate Hatay Hassa Turkey; ^4^ Department of Fruit Sciences ICAR‐CIAH RS Central Horticultural Experiment Station Godhra Gujarat India

**Keywords:** breeding selection, fruit quality traits, genetic resources, local peach accessions, phenotypic variation, quantitative descriptors

## Abstract

Peach ( *Prunus persica* [L.] Batsch) is a vital fruit species in the Rosaceae family, known for its nutritional and commercial importance. Despite its global distribution, modern peach cultivars exhibit limited genetic diversity due to their descent from a few common ancestors. This study assessed the morphological and pomological diversity of 45 native peach accessions from Sistan‐va‐Baluchestan Province, Iran. The evaluation of 46 traits revealed significant phenotypic variability among the accessions. ANOVA showed highly significant differences (*p < 0.05*) for traits like fruit weight (16.62–32.27 g), fruit flesh thickness (7.14–10.50 mm), and total soluble solids (TSS, 11.3%–20.8%). Tree growth habit had the highest coefficient of variation (70.14%), while fruit flesh percentage showed the lowest (3.64%). Correlation analysis revealed strong positive relationships between fruit weight and fruit flesh percentage (*r* = 0.70, *p* < 0.01), while a trade‐off was observed between fruit flesh percentage and TSS (*r* = −0.30, *p* < 0.05). The first three principal components explained 37.65% of the total variation, capturing key patterns in the dataset. Multiple regression analysis (MRA) revealed that fruit weight was significantly influenced by fruit flesh percentage (*β* = 0.91, *p* < 0.00), stone weight (*β* = 0.70, *p* < 0.00), and petal width (*β* = 0.06, *p* < 0.00). TSS was positively associated with stone diameter (*β* = 0.65, *p* < 0.00) but negatively correlated with fruit weight (*β* = −0.38, *p* < 0.01). Among the studied accessions, the first 15 accessions with the highest scores based on individual quantitative datasets were identified as “Panjebog‐19,” “Panjebog‐26,” “Rachedr‐1,” “Panjebog‐20,” “Panjebog‐2,” “Panjebog‐8,” “Panjebog‐24,” “Panjebog‐30,” “Panjebog‐32,” “Panjebog‐10,” “Panjebog‐3,” “Panjebog‐7,” “Panjebog‐33,” “Rachedr‐5,” and “Panjebog‐27.” This study highlights the significant genetic diversity in the studied accessions, showcasing their value for breeding programs. “Panjebog‐19” and “Panjebog‐20,” being among the top 15 superior accessions and positioned outside the 95% confidence ellipse, show potential for developing high‐yielding and high‐quality peach cultivars. Conservation of local germplasm is crucial to counteract urbanization and agricultural pressures. These findings provide a strong foundation for utilizing genetic resources to address challenges in modern peach production, including limited genetic diversity and changing consumer demands, and to promote sustainable breeding strategies. Future studies should focus on integrating molecular markers with morphological and pomological data to improve the understanding of genetic diversity and to develop more targeted breeding strategies.

## Graphical abstract

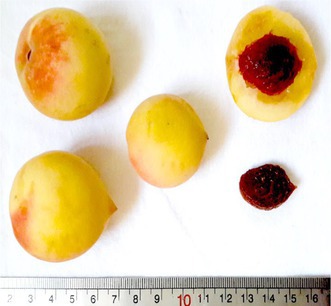

## 1 Introduction

*Prunus* L. is a member of the subfamily Spiraeoideae within the family Rosaceae (Potter et al. 2007). The *Prunus* genus holds significant economic value as a source of fruits, nuts, and timber. It encompasses peaches, plums, cherries, almonds, and apricots. With nearly 200 species, this genus is distributed globally (Chavez et al. 2014).

Peaches ( *Prunus persica* L. Batsch) are among the most widely cultivated stone fruits worldwide, not only valued for their commercial importance but also for their beneficial health effects. Peaches are rich in bioactive compounds including vitamins (especially vitamin C and A), polyphenols, and dietary fiber, which have been linked to antioxidant, anti‐inflammatory, anti‐obesity, and cardioprotective properties, contributing to the prevention of chronic diseases such as cardiovascular disorders and cancer (Bento et al. 2022; Zhao et al. 2023).

In 2022, global peach and nectarine production reached a total of 43.171.565,1 tons. Among the leading producers, the top seven countries were ranked as follows: China (16,800,000 tons), Italy (1,151,490 tons), Türkiye (1,008,185 tons), Greece (894,510 tons), Spain (870,720 tons), the United States of America (666,490 tons), and Iran (577,295 tons) (FAOSTAT 2024).

The peach is thought to have originated in East and Southeast Asia. It spread from China along trade routes through Persia, with the Romans later introducing it across Europe (Scorza and Okie 1991). Alongside this spread, locally adapted peach populations began to develop. Among these are natural populations of vineyard peach in Serbia (Parnia et al. 1988). Peach cultivation on the Balkan Peninsula dates back to between 400 and 300 B.C. Initially propagated from seeds, early peach cultivation over centuries led to the emergence of numerous natural populations characterized by significant variability (Bakić et al. 2017). These populations developed and survived largely independently, adapting to a range of soil and climatic conditions, which resulted in the creation of diverse ecotypes and forms. Vineyard peach, named after its traditional growth in old vineyards, primarily thrived in such environments (Ninkovski 1988). However, yellow‐fleshed varieties eventually gained prominence due to their greater resistance to mechanical damage during harvesting and transport, reducing the significance of traditional vineyard peach in regional production. Additionally, urban expansion, advancements in agriculture, and intensive vineyard management have substantially diminished vineyard peach populations. Therefore, conserving the remaining germplasm of the vineyard peach has become increasingly important.

Genetic diversity is typically categorized into three types: genotypic (based on DNA variation), phenotypic (observable traits), and biochemical (based on metabolites and compounds) (Jiang et al. 2022). Each form of diversity plays a critical role in determining the adaptability and performance of crop species. Morphological and pomological traits, though influenced by environmental conditions, remain key indicators in characterizing phenotypic diversity and are especially useful where molecular resources are limited.

To develop improved peach cultivars, breeders gather a diverse range of cultivars to broaden the gene pool (Maulión et al. 2016; Reig et al. 2013). However, modern peach cultivars exhibit low genetic diversity, as they descend from only a limited number of common ancestors (Myles et al. 2009). The relatively narrow genetic base of the world's dominant peach cultivars has heightened awareness of the need for alternative genetic resources as sources of new genes and alleles (Bakić et al. 2017). Local germplasm represents a valuable foundation for genetic enhancement of the species, and the effective utilization of genetic diversity within these resources at their centers of origin is crucial (Cao et al. 2012). Among these genetic resources is the vineyard peach [ *Prunus persica* (L.) Batsch], a native peach population that either developed naturally or was cultivated in Iran.

The development of new cultivars is crucial for the fruit industry to enhance product quality and captivate consumers with innovative fruit varieties (Infante and Predieri 2008). Modern breeding programs are now centered on meeting the preferences of both producers and consumers. Producers prioritize cultivars that are highly productive, resistant to diseases, and capable of extending the harvest season through varied ripening times, whereas consumers primarily focus on fruit quality attributes (Byrne 2005). Similar to other fruit crops, key factors defining the quality of peach and nectarine fruits include (1) visual qualities like size and skin appearance, (2) sensory qualities, typically assessed by soluble solids concentration and acidity levels, and (3) textural properties, such as firmness (Byrne, Raseira, et al. 2012; Predieri et al. 2005). Consequently, multiple parameters need to be taken into account (Génard and Bruchou 1992), and their interrelations should be explored to enhance fruit assessment methods and breeding practices (Maulión et al. 2016).

Preventing the loss of diversity, preserving potentially valuable traits, and identifying the characters that contribute the most to overall diversity are critically important for plant breeding. To achieve these goals, it is essential to thoroughly examine the morphological and pomological characteristics of peach accessions that develop healthily in their natural environments, remain unaffected by diseases and pests, and meet selection criteria such as high productivity (Barrios‐Masias and Jackson 2014). In this context, significant scientific studies have been conducted worldwide to assess the performance of peach accessions and cultivars (Cantín et al. 2010; I Forcada et al. 2014; Kwon et al. 2015; Reig et al. 2015). However, most of these studies have been limited in exploring the relationships between peach traits in depth due to the insufficient use of multivariate statistical methods. Particularly, studies that use multivariate analyses, which provide more comprehensive and accurate results, are rare (Maulión et al. 2016).

Despite the global relevance of peach breeding, current literature lacks comprehensive evaluations of diverse local germplasm under harsh environmental conditions using integrated multivariate approaches. Most previous studies have either focused on limited trait sets or involved commercially developed cultivars, leaving a substantial gap in understanding the phenotypic performance and breeding potential of native accessions adapted to marginal agro‐ecological zones (Byrne et al. 2012; Cirilli et al. 2021).

This study provides a comprehensive evaluation of 45 indigenous peach accessions exhibiting healthy growth under ecologically challenging conditions, with no visible signs of pest or disease damage. Through the integration of morphological and pomological characterization with multivariate statistical analyses, the research aims to uncover the extent of phenotypic variation and to identify elite accessions with superior agronomic traits. It is hypothesized that multivariate approaches will effectively discriminate promising accessions in terms of productivity, fruit quality, and environmental adaptability. The overarching objective is to enhance breeding strategies by selecting phenotypically diverse and high‐performing accessions suitable for the development of improved peach cultivars.

## 2 Materials and Methods

### 2.1 Plant Material

**FIGURE 1:**
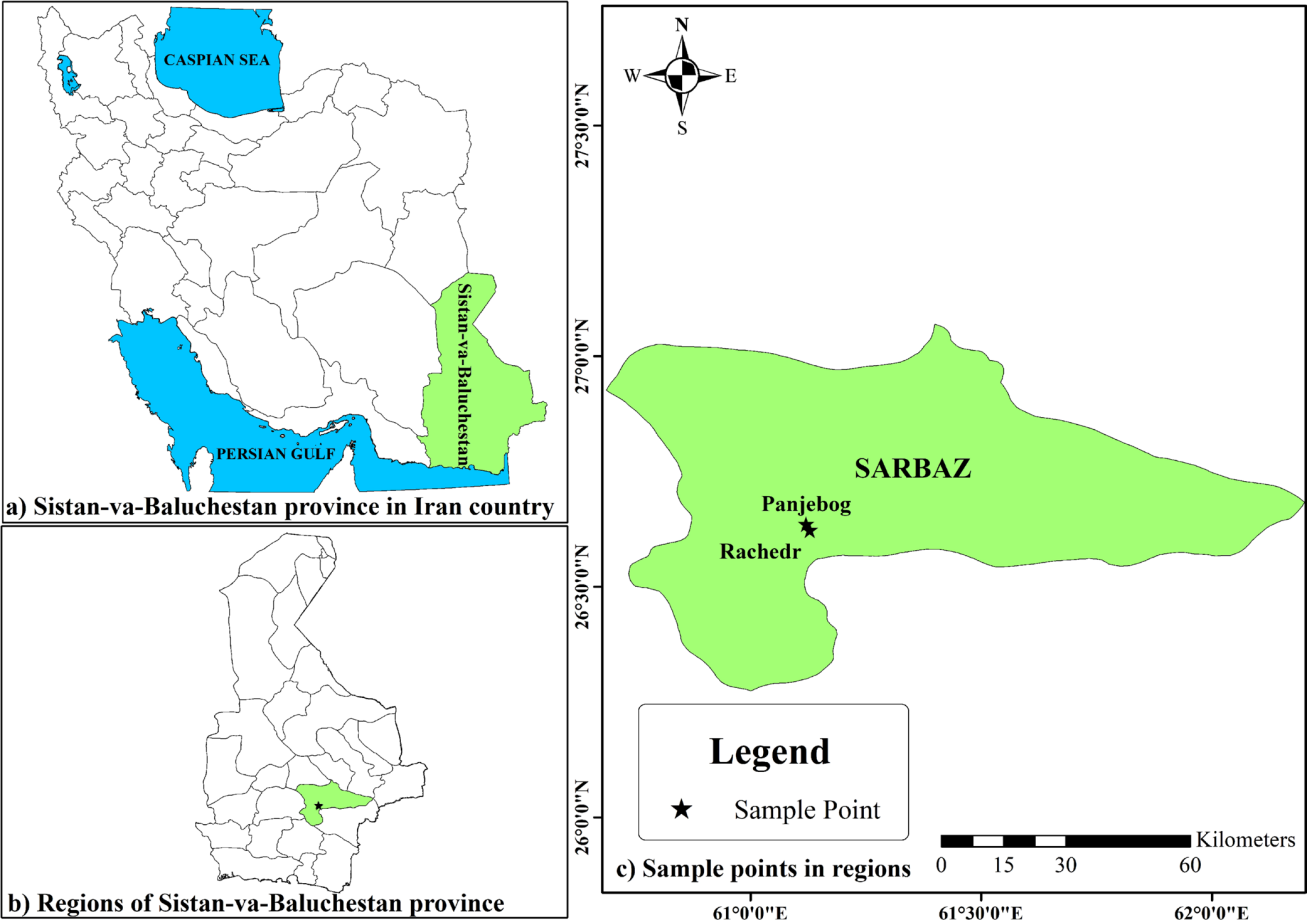
Geographical distribution of the collection sites for the evaluated peach accessions.

Morphological and pomological diversity of 45 native peach accessions was evaluated in two areas of Sistan‐va‐Baluchestan Province, Iran. The geographic locations of collection sites of the studied accessions are shown in Figure 1. For correct sampling, a minimum distance of 200 m was maintained between the accessions in each area to avoid the collection of clonal samples.

### 2.2 Morphological and Pomological Evaluations

Fifty‐two morphological and pomological traits were used to evaluate phenotypic diversity and to select superior accessions (Table 1). The study was conducted using a Completely Randomized Design (CRD) with three replications. In each replication, 10 fully developed flowers, 10 mature leaves, and 10 ripe fruits were randomly selected for each accession. Accordingly, a total of 30 flowers, 30 leaves, and 30 fruits per accession were evaluated. Morphological and pomological traits related to the dimensions of flowers, leaves, fruits, and stones were precisely measured using a digital caliper (Loyka B5110‐150, IP54 certified, China) with a resolution of 0.01 mm to ensure measurement accuracy (Șumălan et al. 2025). Fruit and stone weights were recorded using a precision electronic balance (JNB5002, JOANLAB, Zhejiang, China) with a sensitivity of 0.01 g (Colak et al. 2025). Total soluble solids content was determined in freshly extracted fruit juice using a digital refractometer (Pocket PAL‐1, ATAGO Corporation, Tokyo, Japan) and expressed as degrees Brix (°Bx) (Wang et al. 2024). All measurements were conducted under consistent laboratory conditions to minimize experimental variability. The qualitative traits (Table 2) were visually examined and coded based on the peach descriptor (IBPGR and CEC 1984).

**TABLE 1:** Descriptive statistical parameters of the quantitative pomological and morphological traits recorded in the peach accessions.

Trait	Abb	Unit	Min	Max	Mean	*±*SD	CV (%)
Tree growth habit	V1	Code	1	5	2.07	1.45	70.14
Tree growth vigor	V2	Code	1	5	3.22	1.55	48.14
Tree height	V3	Code	1	5	3.00	1.28	42.63
Trunk type	V4	Code	1	7	4.60	2.16	46.89
Canopy density	V5	Code	1	5	3.22	1.43	44.35
Branching	V6	Code	1	5	2.87	1.38	47.91
Flowering date	V7	Code	1	5	2.78	1.22	43.99
Flower density	V8	Code	1	5	3.36	1.37	40.71
Peduncle length	V9	mm	2.18	5.54	3.52	0.72	20.53
Peduncle width	V10	mm	1.35	3.00	2.17	0.33	15.24
Petal shape	V11	Code	1	3	1.27	0.69	54.17
Petal length	V12	mm	14.84	23.41	18.49	1.84	9.96
Petal width	V13	mm	13.02	19.96	16.08	1.54	9.56
Hypanthium length	V14	mm	2.52	5.90	4.06	0.75	18.49
Hypanthium diameter	V15	mm	2.42	5.11	4.03	0.59	14.71
Sepal external color	V16	Code	1	3	2.11	1.01	47.63
Sepal length	V17	mm	3.24	7.30	6.01	0.82	13.60
Sepal width	V18	mm	3.42	5.73	4.69	0.59	12.65
Number of stamens	V19	Number	24	50	35.13	4.34	12.36
Length of the stamen in relation to carpel	V20	Code	1	3	1.40	0.81	57.79
Leaf length	V21	mm	54.25	96.94	74.90	10.55	14.08
Leaf width	V22	mm	15.68	23.63	20.46	1.76	8.60
Petiole length	V23	mm	4.60	8.05	6.27	0.92	14.71
Petiole diameter	V24	mm	0.59	1.29	0.87	0.12	13.71
Leaf shape	V25	Code	1	3	2.47	0.89	36.19
Leaf margin form	V26	Code	1	3	2.33	0.95	40.90
Leaf serration shape	V27	Code	1	3	1.76	0.98	55.74
Leaf color	V28	Code	1	5	2.20	1.24	56.18
Leaf apex shape	V29	Code	1	5	2.47	1.56	63.20
Ripening date	V30	Code	1	5	3.00	1.04	34.80
Fruit yield	V31	Code	1	5	3.18	1.27	39.84
Fruit length	V32	mm	32.22	43.65	37.46	2.80	7.47
Fruit width	V33	mm	30.54	38.88	34.49	2.12	6.16
Fruit diameter	V34	mm	28.45	39.12	33.19	2.62	7.91
Fruit weight	V35	g	16.62	32.27	23.84	4.69	19.66
Fruit skin blush color	V36	Code	1	5	2.91	1.65	56.67
Fruit flesh thickness	V37	mm	7.14	10.50	8.87	0.83	9.37
Fruit flesh color	V38	Code	1	5	2.60	1.25	48.08
Fruit flesh percentage	V39	%	78.57	90.61	84.29	3.07	3.64
Depth of fruit stalk cavity	V40	mm	2.94	5.85	3.99	0.70	17.41
Fruit stalk attachment diameter	V41	mm	3.24	5.17	4.06	0.42	10.44
Stone length	V42	mm	20.12	35.08	23.80	2.46	10.34
Stone width	V43	mm	14.72	20.47	18.06	1.43	7.92
Stone diameter	V44	mm	11.08	15.94	13.79	1.19	8.62
Stone weight	V45	g	2.62	5.17	3.65	0.60	16.46
Total soluble solids	V46	%	11.30	20.80	15.48	2.07	13.35

Abbreviations: ±SD, standard deviation; Abb, abbreviations; CV, coefficient of variation; Max, maximum; Min, minimum.

**TABLE 2:** Frequency distribution of qualitative pomological and morphological traits among the evaluated peach accessions.

Traits	1	3	5	7
Tree growth habit	Spreading (27)	Open (12)	Semi‐erect (6)	—
Tree growth vigor	Low (11)	Moderate (18)	High (16)	—
Tree height	Low (9)	Moderate (27)	High (9)	—
Trunk type	Single trunk (7)	Multi‐trunk, low (10)	Multi‐trunk, moderate (13)	Multi‐trunk, high (15)
Canopy density	Low (9)	Moderate (22)	High (14)	—
Branching	Low (12)	Moderate (24)	High (9)	—
Flowering date	Mid February (11)	Late February (28)	Early March (6)	—
Flower density	Low (7)	Moderate (23)	High (15)	—
Petal shape	Round (39)	Obovat (6)	—	—
Sepal external color	Green‐crimson (20)	Crimson (25)	—	—
Length of the stamen in relation to carpel	Equal (36)	Longer (9)	—	—
Leaf shape	Lanceolate (12)	Oblanceolate (33)	—	—
Form of leaf margin	Entire (15)	Wavy (30)	—	—
Leaf serration shape	Serrate (28)	Crenate (17)	—	—
Leaf color	Light green (21)	Green (21)	Dark green (3)	—
Leaf apex shape	Caudate (21)	Acute (15)	Broadly acute (9)	—
Ripening date	Late May (6)	Early June (33)	Mid June (6)	—
Fruit yield	Low (7)	Moderate (27)	High (11)	—
Fruit skin blush	Yellow (16)	Light red (15)	Red (14)	—
Fruit flesh color	Light yellow (14)	Yellow (26)	Yellow‐red (5)	—

### 2.3 Statistical Analysis

Analysis of variance (One‐way ANOVA, *p < 0.05*) was performed to evaluate the variation among accessions based on the traits measured using JMP Pro 17 software (JMP 2024). Pearson (*r*) correlation coefficients were used to determine the relationship between the recorded traits using Origin Pro 2024b software (OriginLab 2024). Principal component analysis (PCA) was applied using Pro 2024b software to identify the key traits influencing genotype grouping. To enhance the interpretability of the components, the Varimax rotation with the Kaiser Normalization method was employed. This approach clarified the relationships between components, ensuring a more comprehensible and meaningful analysis. Heat map analysis based on Ward's method and Euclidean distance coefficients using Origin Pro 2024b software was used to classify accessions and variables. The first and second principal components (PC1/PC2) were used to draw a two‐dimensional biplot by determining the distribution of accessions and quantitative variables using Origin Pro 2024b software. Moreover, fruit‐related traits were considered dependent variables, and the characteristics affecting these characters were determined using multiple regression analysis (MRA). The MRA was conducted using “stepwise” method of “linear regression analysis” option of SPSS (SPSS Inc., Chicago, IL, USA) (Efe et al. 2000; Norusis 1998) statistics.

## 3 Results and Discussion

### 3.1 Descriptive Statistics Among Accessions

Descriptive statistics for the pomological and morphological traits utilized in the studied peach accessions are presented in detail in Table 1. Goodarzi et al. (2018), Mostafa et al. (2020), Elwakil et al. (2021), and Khadivi et al. (2022) reported that certain variables did not show significant differences among the accessions (CV = 0.00%) in their studies, leading them to exclude these variables from their analyses. As a result, since six variables (petal color, petal number, petal apex shape, hypanthium shape, fruit pubescence, and fruit skin ground color) did not show a significant difference (CV = 0.00%) among the accessions, the evaluations were made based on 46 variables. In this context, a one‐way analysis of variance (ANOVA) was performed to evaluate the statistical significance of differences among the peach accessions for each measured trait. The analysis, conducted at a 95% confidence level (*p* < 0.05), revealed highly significant variations among the accessions, indicating a considerable degree of phenotypic diversity within the studied population. Following ANOVA, Tukey's Honestly Significant Difference (HSD) test was applied to determine pairwise differences among accession means. The highest variation was observed in tree growth habit (70.14%), leaf apex shape (63.20%), length of the stamen in relation to carpel (57.79%) fruit skin blush color (56.67%), and leaf color (56.18%). In contrast, the lowest variation was recorded in stone width (7.92%), fruit diameter (7.91%), fruit length (7.47%), fruit width (6.16%), and fruit flesh percentage (3.64%). Notably, 21 out of 46 variables (representing 45.65% in total) had coefficients of variation (CVs) greater than 20.00%. Our findings are consistent with a similar study conducted in Iran, which reported a CV of 47.83% (Khadivi et al. 2024). This value indicates a high degree of variation among the examined accessions (Khadivi et al. 2024).

Peduncle length ranged from 2.18 (“Panjebog‐12”) to 5.54 (“Panjebog‐28”) mm, peduncle width changed between 1.35 (“Panjebog‐10”) and 3.00 (“Panjebog‐7”) mm, petal length varied between 14.84 (“Rachedr‐9”) and 23.41 (“Panjebog‐19”) mm, petal width ranged from 13.02 (“Rachedr‐2”) to 19.96 (“Panjebog‐27”) mm, hypanthium length changed between 2.52 (“Panjebog‐1”) and 5.90 (“Panjebog‐33”) mm, hypanthium diameter varied between 2.42 (“Rachedr‐12”) and 5.11 (“Panjebog‐19”) mm, sepal length ranged from 3.24 (“Panjebog‐8”) to 7.30 (“Panjebog‐9”) mm, sepal width changed between 3.42 (“Rachedr‐8”) and 5.73 (“Rachedr‐7”) mm, number of stamens varied between 24 (“Rachedr‐6”) and 50 (“Rachedr‐5”), leaf length ranged from 54.25 (“Rachedr‐9”) to 96.94 (“Panjebog‐19”) mm, leaf width changed between 15.68 (“Panjebog‐14”) and 23.63 (“Panjebog‐29”) mm, petiole length varied between 4.60 (“Panjebog‐5”) and 8.05 (“Rachedr‐10”) mm, petiole diameter ranged from 0.59 (“Panjebog‐14”) to 1.29 (“Panjebog‐30”) mm, fruit length changed between 32.22 (“Panjebog‐22”) and 43.65 (“Panjebog‐26”) mm, fruit width varied between 30.54 (“Panjebog‐22”) and 38.88 (“Panjebog‐8”) mm, fruit diameter ranged from 28.45 (“Panjebog‐22”) to 39.12 (“Panjebog‐8”) mm, fruit weight changed between 16.62 (“Rachedr‐9”) and 32.27 (“Panjebog‐8”) g, fruit flesh thickness varied between 7.14 (“Rachedr‐17”) and 10.50 (“Rachedr‐8”) mm, fruit flesh percentage ranged from 78.57% (“Rachedr‐23”) to 90.61% (“Rachedr‐20”), depth of fruit stalk cavity changed between 2.94 (“Rachedr‐17”) and 5.85 (“Rachedr‐24”) mm, fruit stalk attachment diameter varied between 3.24 (“Rachedr‐14”) and 5.17 (“Rachedr‐8”) mm, stone length ranged from 20.12 (“Rachedr‐22”) to 35.08 (“Rachedr‐2”) mm, stone width changed between 14.72 (“Rachedr‐4”) and 20.47 (“Rachedr‐24”) mm, stone diameter varied between 11.08 (“Rachedr‐4”) and 15.94 (“Rachedr‐13”) mm, stone weight ranged from 2.62 (“Rachedr‐20”) to 5.17 (“Rachedr‐24”) g, and total soluble solids changed between 11.3% (“Rachedr‐2”) and 20.8% (“Rachedr‐5”).

Paunovic et al. (1990) reported that fruit weight ranged from 15.84 to 68.70 g. In a similar study conducted in Spain, total soluble solids changed between 9.5°Bx and 18.6°Bx (Reig et al. 2015), while in Greece, these values varied between 9.01°Bx and 16.6°Bx (Drogoudi et al. 2023). Milosevic and Milosevic (2010) identified fruit weight as ranging from 16.62 to 32.27 g and stone weight as changing between 2.62 and 5.17 g. Similarly, a study conducted in Serbia reported petal length ranging from 14.84 to 23.41 mm, petal width changing between 13.02 and 19.96 mm, number of stamens varying between 24 and 50, leaf length ranging from 54.25 to 96.94 mm, leaf width changing between 15.68 and 23.63 mm, petiole length varying between 4.60 and 8.05 mm, stone width ranging from 14.72 to 20.47 mm, and total soluble solids changing between 11.30°Bx and 20.80°Bx (Bakić et al. 2017).

Our findings are generally in line with those of previous researchers. Minor differences observed may be attributed to variations in environmental conditions or genetic diversity among accessions (Crawford and Whitney 2010).

Frequency distribution is a technique used in the analysis of qualitative data and shows how frequently each category or class occurs within the dataset. Frequency distribution helps to identify which categories are more common and which are less represented. The frequencies of categorical data provide valuable information for researchers to understand the structure and distribution of the data. As a result, frequency distribution is a fundamental tool for organizing and interpreting qualitative data (Bryman 2016).

The frequency distribution of the measured qualitative pomological and morphological characteristics in the peach accessions studied is presented in detail in Table 2. The morphological and pomological traits of the studied peach accessions displayed considerable diversity. The variation in tree growth habit is noteworthy, with the majority of the accessions exhibiting a spreading growth habit (27 accessions), followed by open (12) and semi‐erect (6). This suggests that a predominantly spreading growth habit is common in the studied population. This trait has practical implications for orchard spacing and management, as spreading trees may require more space to grow and may have a greater need for pruning to maintain optimal light penetration and air circulation. On the other hand, the fewer semi‐erect trees may present more compact growth forms, potentially beneficial for high‐density orcharding systems where space is limited.

Tree growth vigor is another important trait, with moderate vigor being the most frequent (18 accessions), followed by high vigor (16) and low vigor (11). Moderate vigor is often considered ideal for peach cultivation, as it balances growth and fruit production, preventing excessive vegetative growth that could limit fruit yield. However, accessions with high vigor may require more intensive management practices, such as regular pruning and nutrient management, to control excessive canopy growth and ensure that energy is directed toward fruiting rather than vegetative expansion.

The variation in tree height is similarly significant. The majority of accessions were of moderate height (27), which is advantageous for ease of harvesting and overall orchard management. While shorter trees (9 accessions) may facilitate harvesting and require less labor, taller trees (9) could potentially yield more fruit if properly managed, though they may pose challenges in terms of harvesting efficiency and susceptibility to wind damage.

The data on trunk type indicate considerable diversity, with multi‐trunk trees being the most frequent, especially those with moderate (13) and high (15) numbers of trunks. Multi‐trunk trees can provide greater structural stability, which is particularly important in regions with strong winds. However, the presence of single‐trunk trees (7) may allow for more direct access to fruit and simpler pruning practices, which could be an advantage in certain production systems.

Canopy density also shows considerable variability. Most accessions exhibited moderate canopy density (22), which is generally ideal for light penetration and air circulation, reducing the risk of fungal diseases. High canopy density (14) might indicate a more productive tree but could also increase the risk of poor air circulation and increased disease susceptibility. Low canopy density (9), while offering better airflow, may lead to reduced fruit set due to insufficient leaf area for photosynthesis.

The timing of flowering is an important factor in determining the potential for frost damage and the suitability of the accessions for different growing regions. Late February (28 accessions) was the most common flowering date, followed by mid‐February (11) and early March (6). Accessions flowering later may be less prone to early frost damage, which can be a significant risk in temperate climates.

Flower density is another trait that exhibited substantial variability. Moderate flower density (23 accessions) is typical for many peach varieties, as it allows for a balanced fruit set without the risk of excessive thinning. High flower density (15 accessions) could indicate higher potential yields but may also require more rigorous thinning to ensure optimal fruit size and quality.

The petal shape and sepal color results also suggest interesting diversity in aesthetic traits. The majority of accessions had round petals (39), which are generally associated with higher‐quality blooms and potentially greater attractiveness to pollinators. The variation in sepal external color, with crimson being the dominant hue (25 accessions), could have implications for the marketability of the fruit, as color is often a significant factor in consumer preferences.

The length of the stamen relative to the carpel was mostly equal (36 accessions), which is a typical characteristic for self‐fertile varieties. However, the presence of longer stamens (9 accessions) could indicate a possibility for cross‐pollination, potentially contributing to genetic diversity within orchard populations.

Leaf shape, margin form, and serration type further highlight the phenotypic diversity within the accessions. Oblanceolate leaves (33 accessions) are typical of many peach varieties and are generally well‐suited for optimal light interception. Wavy leaf margins (30 accessions) could indicate greater resistance to certain environmental stresses, such as wind or drought, while serrate leaf margins (28 accessions) are a characteristic of many fruit‐bearing species, potentially contributing to increased photosynthetic capacity.

The variation in leaf color, with the majority of accessions exhibiting green leaves (21), suggests that this is a common trait among the studied accessions. However, the presence of both light green (21) and dark green (3) leaves may reflect genetic variation in chlorophyll content, which can influence photosynthetic efficiency and overall tree vigor.

The leaf apex shape, with a predominance of caudate leaves (21 accessions), may be related to environmental adaptation, as this shape could be more resistant to certain pest attacks. The broader acute (9 accessions) and acute (15 accessions) shapes suggest that different accessions may have evolved under varying environmental pressures.

The ripening date is a key trait for market timing and storage potential, with early June (33 accessions) being the most common ripening period. This trait is particularly important for commercial producers seeking to optimize harvest windows and market supply. Late May (6 accessions) and mid‐June (6) ripening accessions may offer extended harvest periods, potentially spreading the workload and reducing the risk of crop loss due to late‐season weather events.

Finally, the variation in fruit yield and skin blush color reflects the diversity in the genetic potential for fruit production. Moderate yield (27 accessions) is common and is typically the most sustainable in terms of orchard management. High yield (11 accessions: “Panjebog‐2,” “Panjebog‐6,” “Panjebog‐7,” “Panjebog‐8,” “Panjebog‐19,” “Panjebog‐20,” “Panjebog‐21,” “Panjebog‐30,” “Rachedr‐3,” “Rachedr‐7,” and “Rachedr‐11”) may provide greater returns but could require more intensive management. The blush color of fruit, with a predominance of yellow (16 accessions), light red (15), and red (14), further highlights the diversity in aesthetic qualities, which could influence consumer preference and marketability.

When our findings are evaluated alongside those of similar studies conducted in France (Quilot et al. 2004) and Greece (Drogoudi et al. 2023), notable differences emerge. These differences can be attributed to ecological factors and the genetic variability of the accessions used.

**FIGURE 2:**
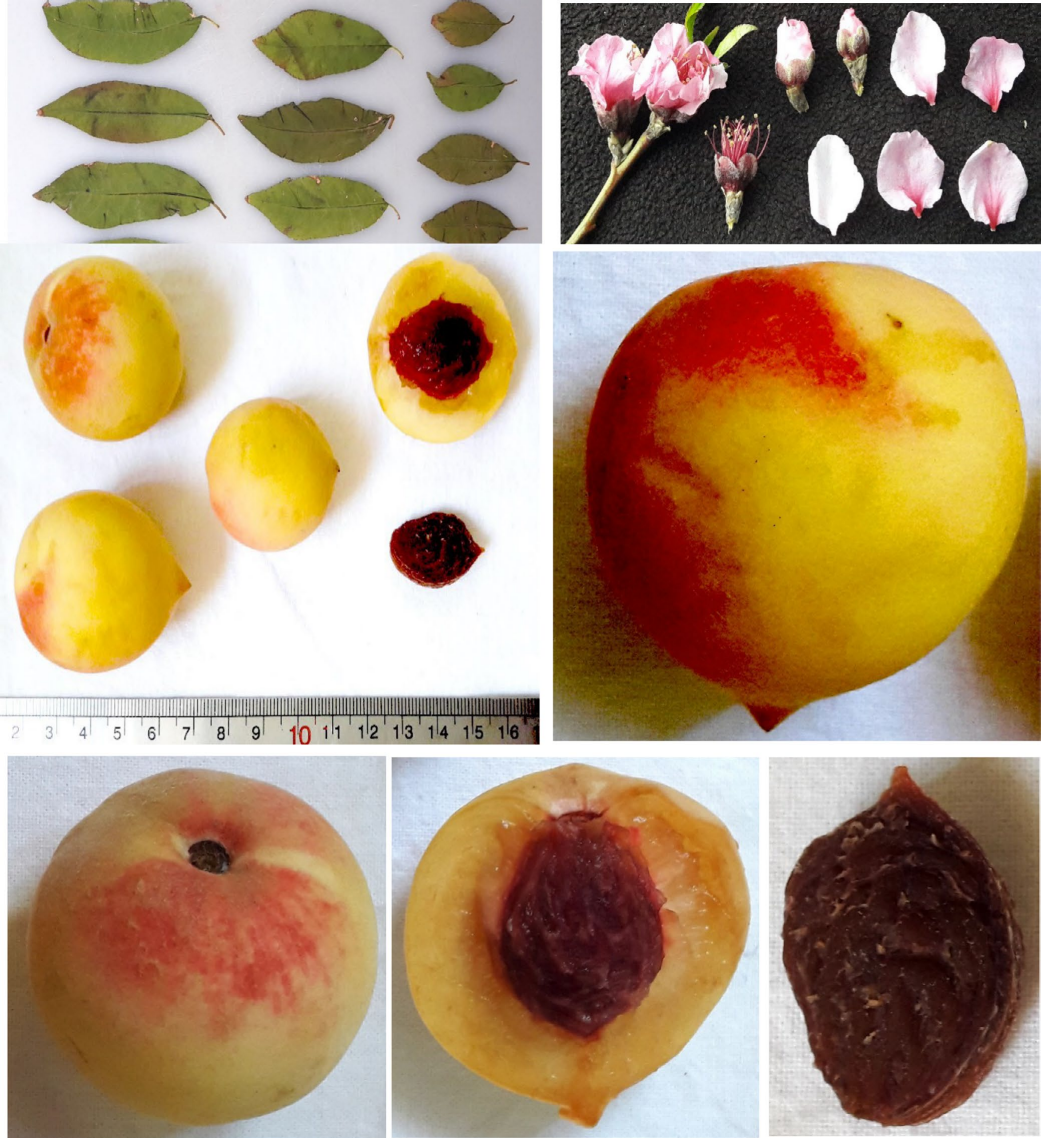
Representative flower, leaf, and fruit morphology of the studied peach accessions.

In conclusion, the morphological and pomological characteristics of the studied peach accessions show considerable variation, which can be leveraged for breeding programs focused on improving traits such as tree vigor, fruit yield, and disease resistance. The diversity observed in these accessions suggests that there is significant potential for selecting individuals with desirable traits for specific growing conditions and market demands. The flower, leaf, and fruit of the studied peach accessions are shown in Figure 2.

### 3.2 Correlation Matrix Analysis (CMA)

Correlation matrix analysis (CMA) is a statistical technique that allows for the systematic examination of linear relationships between multiple variables. This analysis determines the strength and direction of the relationship between variables by calculating the Pearson correlation coefficient (*r*) for each pair of variables. The Pearson correlation coefficient (*r*) is the most commonly used method for measuring the intensity of the linear relationship between two continuous variables and takes a value between −1 and 1 (Pearson 1900). This coefficient defines the relationship between variables linearly; a value of 1 indicates a perfect positive linear relationship, −1 indicates a perfect negative linear relationship, and 0 indicates no linear relationship. The Pearson correlation coefficient (*r*) is highly effective in identifying linear relationships.

This matrix provides a visual summary of the overall relationships in the data. Such an analysis is particularly important when dealing with a large number of variables and understanding the interactions between them (Tabachnick et al. 2013). This analysis is used when working with quantitative data (Creswell and Creswell 2017).

**FIGURE 3:**
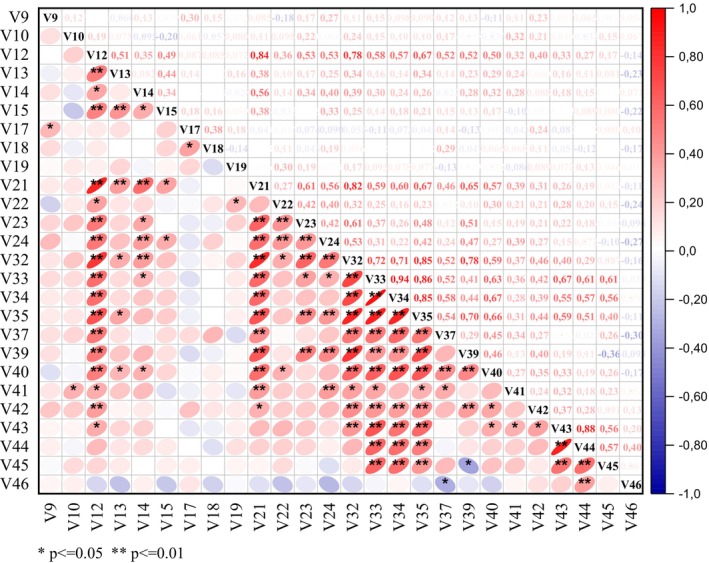
Pearson correlation matrix illustrating the relationships among quantitative pomological and morphological traits in peach accessions (for abbreviations, see Table 1).

Accordingly, the simple correlations among the quantitative pomological and morphological variables used in the studied peach accessions are presented in Figure 3. Among the observed traits, significant positive correlations were noted between petal length and leaf length (*r* = 0.84, *p* < 0.01), suggesting that larger petals may coincide with longer leaves. This could reflect a coordinated growth mechanism influenced by genetic or environmental factors (Brock and Weinig 2007). Similarly, petiole length also exhibited a strong correlation with leaf length (*r* = 0.61, *p* < 0.01) and petiole diameter (*r* = 0.56 *p* < 0.01), indicating an integrated relationship between leaf size and its supporting structures (Lal et al. 2019).

Fruit‐related characteristics demonstrated strong interrelationships. For instance, fruit length exhibited a high positive correlation with fruit weight (*r* = 0.85, *p* < 0.01), fruit flesh thickness (*r* = 0.52, *p* < 0.01), and stone length (*r* = 0.46, *p* < 0.01), indicating that as fruit size increases, both internal tissue development and stone dimensions tend to scale proportionally. Furthermore, fruit diameter and fruit width showed a remarkably strong association (*r* = 0.94, *p* < 0.01), underscoring the geometric symmetry inherent in fruit morphology (Ghahremani et al. 2023).

Petal length was also positively associated with both fruit length (*r* = 0.78, *p* < 0.01) and fruit weight (*r* = 0.68, *p* < 0.01), suggesting that floral organ size may serve as an indirect indicator of fruit development, potentially reflecting overall plant vigor and resource allocation patterns (Brock and Weinig 2007).

Leaf traits further reinforced their contribution to fruit formation. Leaf length correlated significantly with fruit length (*r* = 0.82, *p* < 0.01) and fruit weight (*r* = 0.68, *p* < 0.01), implying that larger leaves‐hich may indicate enhanced photosynthetic capacity‐re linked to increased fruit growth and biomass accumulation (Flore and Lakso 1989).

Additionally, stone length maintained a moderate positive correlation with fruit length (*r* = 0.46, *p* < 0.01), suggesting that accessions producing larger fruits also tend to develop proportionally larger stones. Similarly, stone weight correlated positively with both stone diameter (*r* = 0.57, *p* < 0.01) and fruit diameter (*r* = 0.56, *p* < 0.01), highlighting a consistent scaling relationship between stone and fruit dimensions. However, the relationship between stone weight and total soluble solids was comparatively weaker (*r* = 0.40, *p* < 0.01), indicating that stone size is not a reliable predictor of fruit sweetness or sugar content (Flore 2018).

A strong positive association was also found between fruit flesh percentage and fruit weight (*r* = 0.70, *p* < 0.01), suggesting that increases in edible tissue proportion contribute directly to heavier fruit mass. This correlation has important implications for breeding, as selecting genotypes with higher flesh content can indirectly enhance total fruit weight—an essential commercial attribute. From a practical standpoint, this relationship may inform thinning and orchard management strategies that prioritize fruits with superior flesh yield, thereby improving both productivity and market value. Nevertheless, the strength of this association may vary depending on genotypic background and environmental conditions, necessitating a context‐specific approach in breeding and cultivation programs (Núñez‐Lillo et al. 2024).

The diameter of the hypanthium showed moderate yet statistically significant correlations with both petal width (*r* = 0.44, *p* < 0.01) and petal length (*r* = 0.49, *p* < 0.01), suggesting that flowers with larger hypanthia may possess more prominent petals, which could potentially enhance pollination efficiency and reproductive success (Brock and Weinig 2007).

Fruit flesh thickness was also positively correlated with the depth of the fruit stalk cavity (*r* = 0.45, *p* < 0.01), indicating that increased flesh thickness may coincide with deeper stalk cavities–possibly due to shared structural constraints or developmental pathways during fruit maturation.

Finally, a significant positive correlation was observed between peduncle width and fruit stalk attachment diameter (*r* = 0.32, *p* < 0.05), implying that thicker peduncles may offer improved support for fruit attachment. This trait could play a critical role in enhancing mechanical stability and reducing fruit drop during development and harvesting operations (Taskin 2017).

The findings highlight the tightly regulated developmental relationships among vegetative, floral, and fruit traits in peach accessions. The strong correlations between leaf traits and fruit size underscore the critical role of vegetative growth in supporting reproductive output. Simultaneously, the interplay between petal and hypanthium dimensions suggests that floral architecture is tightly coordinated, likely optimizing reproductive efficiency.

The trade‐off between fruit flesh percentage and total soluble solids remains a key breeding consideration, as enhancing sweetness may reduce juiciness or flesh content. Moreover, the associations between peduncle width and fruit attachment characteristics suggest an avenue for improving fruit retention and reducing pre‐harvest losses.

Overall, the study provides a comprehensive framework for understanding the interconnected nature of morphological and pomological traits in peach accessions. These insights have practical implications for breeding strategies focused on yield, quality, and adaptability while addressing potential trade‐offs between key fruit attributes. Our findings are consistent with the results of previous studies in the field.

### 3.3 Multiple Regression Analysis (MRA)

Multiple regression analysis (MRA) is a statistical method used to assess the relationship between a dependent variable and multiple independent variables. It helps to understand how several predictors influence the outcome. The dependent variable is the one being predicted, while the independent variables are those believed to influence the dependent variable. Key components of MRA include the correlation coefficient (*r*), which measures the strength and direction of the relationship, and *r* ^2^ (the coefficient of determination), which shows how much of the dependent variable's variability is explained by the independent variables. A higher *r* ^2^ indicates a better fit of the model. The *β* (beta coefficient) quantifies the effect of each independent variable on the dependent variable, while the *t*‐value tests the significance of each coefficient. The *p*‐value determines if the effect of a predictor is statistically significant, with values below 0.05 typically indicating significance. Overall, MRA allows researchers to quantify relationships between variables, assess the impact of each predictor, and make predictions, making it an essential tool in many fields (Cohen et al. 2013; Hair et al. 2010; Montgomery et al. 2021).

Initially, after calculating the simple correlation coefficients, fruit weight, fruit flesh thickness, fruit flesh percentage, stone weight, and total soluble solids were considered as dependent variables, and the direct and indirect effects of the independent variables on these key traits were examined. The results of the MRA revealed that fruit weight was associated with four traits (fruit flesh percentage, stone weight, fruit flesh thickness, and petal width), fruit flesh thickness with three traits (fruit diameter, stone diameter, and sepal width), fruit flesh percentage with three traits (stone weight, fruit weight, and total soluble solids), stone weight with five traits (fruit width, fruit flesh percentage, fruit weight, total soluble solids, and fruit flesh thickness), total soluble solids with three traits (stone diameter, fruit weight, and leaf width) (Table 3).

**TABLE 3:** Stepwise multiple regression analysis (MRA) identifying key pomological traits contributing to fruit‐related characteristics in the peach accessions.

Dependent character	Independent character	*r*	*r* ^*2*^	*β*	*t*	*p*
Fruit weight	Fruit flesh percentage	0.860a	0.74	**0.91**	36.67	**0.00** *
	Stone weight	0.941b	0.89	**0.70**	28.92	**0.00** *
	Fruit flesh thickness	0.987c	0.98	**0.08**	3.52	**0.00** *
	Petal width	0.987d	0.98	**0.06**	3.02	**0.00** *
Fruit flesh thickness	Fruit diameter	0.581a	0.34	**0.85**	6.78	**0.00** *
	Stone diameter	0.721b	0.52	−0.48	−3.78	0.00
	Sepal width	0.753c	0.57	0.22	2.14	0.04
Fruit flesh percentage	Stone weight	0.784a	0.61	**−0.77**	−30.21	**0.00** *
	Fruit weight	0.893b	0.80	**1.02**	39.74	**0.00** *
	Total soluble solids	0.987c	0.97	0.07	2.91	0.01
Stone weight	Fruit width	0.612a	0.37	**0.14**	2.14	**0.04** *
	Fruit flesh percentage	0.905b	0.82	**−1.21**	−26.46	**0.00** *
	Fruit weight	0.980c	0.96	**1.17**	14.08	**0.00** *
	Total soluble solids	0.983d	0.97	0.07	2.22	0.03
	Fruit flesh thickness	0.985e	0.97	−0.07	−2.03	0.05
Total soluble solids	Stone diameter	0.399a	0.16	**0.65**	4.44	**0.00** *
	Fruit weight	0.542b	0.29	−0.38	−2.58	0.01
	Leaf width	0.604c	0.37	−0.28	−2.14	0.04

* Multiple regression analysis correlations are supported by the correlation matrix analysis. Bold values are statistically significant *p* < 0.01.

The strong positive association between fruit weight and fruit flesh percentage (*β* = 0.91, *p* < 0.00) underscores the critical role of flesh content in determining the overall mass of the fruit. This suggests that breeding programs aiming to increase fruit weight should prioritize improvements in flesh percentage, as it directly contributes to consumer preference and market value. The significant positive contributions of stone weight (*β* = 0.70, *p* < 0.00), fruit flesh thickness (*β* = 0.08, *p* < 0.00), and petal width (*β* = 0.06, *p* < 0.00) further highlight the multifaceted influences on fruit weight, suggesting that both internal (e.g., stone weight) and external (e.g., petal width) traits work in synergy to shape fruit morphology.

The relationship between fruit flesh thickness and fruit diameter (*β* = 0.85, *p* < 0.00) demonstrates a logical association, as larger fruit diameters naturally allow for greater flesh accumulation. However, the negative effect of stone diameter on flesh thickness (*β* = −0.48, *p* < 0.00) points to a potential spatial trade‐off within the fruit structure, where an increase in stone size may limit the space available for flesh development. The positive but weaker influence of sepal width (*β* = 0.22, *p* < 0.04) suggests that floral characteristics may also play a role in determining fruit morphology, albeit to a lesser degree.

Fruit flesh percentage exhibited contrasting relationships with stone weight (negative) and fruit weight (positive). The strong negative association with stone weight (*β* = −0.77, *p* < 0.00) implies that as stones become heavier, they proportionally reduce the relative flesh content. Conversely, the strong positive effect of fruit weight (*β* = 1.02, *p* < 0.00) highlights that larger fruits tend to have a higher flesh proportion, possibly due to optimized resource allocation toward edible parts of the fruit. Interestingly, total soluble solids had a minor positive effect on flesh percentage (*β* = 0.07, *p* < 0.01), suggesting that sugar concentration, while not a major determinant of flesh proportion, could still contribute marginally to consumer perception of fruit quality.

The analysis of stone weight reveals complex interactions. While fruit weight (*β* = 1.17, *p* < 0.00) and fruit width (*β* = 0.14, *p* < 0.04) positively influenced stone weight, the strong negative association with fruit flesh percentage (*β* = −1.21, *p* < 0.00) highlights a trade‐off between stone development and flesh allocation. This finding is particularly relevant for breeding programs, as an excessive focus on increasing fruit weight could inadvertently lead to larger stones, which may be undesirable from a consumer perspective. Additionally, the weak negative influence of fruit flesh thickness on stone weight (*β* = −0.07, *p* < 0.05) further supports the idea of a competitive relationship between these two traits. Interestingly, stone weight was also found to have a positive relationship with total soluble solids (*β* = 0.07, *p* < 0.03), suggesting that larger stones may be associated with increased sugar concentrations in the fruit. This finding highlights a potential advantage of larger stones in contributing to fruit sweetness, which may partially offset their drawbacks, depending on the breeding goals and consumer preferences.

Total soluble solids showed a significant positive relationship with stone diameter (*β* = 0.65, *p* < 0.00), suggesting that larger stones may correlate with higher sugar concentrations. However, the negative effects of fruit weight (*β* = −0.38, *p* < 0.01) and leaf width (*β* = −0.28, *p* < 0.04) indicate a potential dilution effect in larger fruits, where an increase in fruit size may result in lower relative sugar concentration. This finding underscores the challenge of balancing size and sweetness in breeding programs. The negative influence of leaf width on TSS could reflect a physiological trade‐off, where broader leaves may prioritize vegetative growth over the allocation of resources to fruit sugar accumulation. The bold values are supported by the correlation matrix analysis.

To the best of our knowledge, MRA has not been applied previously in studies focusing on the morphological and pomological traits of peach accessions. Therefore, the MRA findings in this study have been extensively and independently discussed to provide a comprehensive understanding of the interdependencies among these traits. This analytical approach offers a novel perspective to the existing literature, contributing unique insights into how key fruit characteristics interact at a morphological and biochemical level. Such detailed discussions help bridge existing gaps in knowledge and may inspire future research to build upon these findings, particularly in the context of optimizing fruit quality through targeted breeding strategies.

In summary, the results reveal a series of trade‐offs and synergies among peach fruit traits, reflecting the complexity of their biological and genetic regulation. Breeding programs must carefully navigate these relationships, as optimizing one trait may have unintended consequences for others. For example, efforts to increase fruit weight should be accompanied by strategies to control stone size and maintain high flesh percentages. Similarly, enhancing total soluble solids must account for potential size‐related dilution effects. These findings emphasize the importance of holistic breeding approaches that consider the interdependencies of multiple traits to achieve balanced improvements in fruit quality and consumer appeal.

### 3.4 Principal Component Analysis (PCA)

In this study, principal component analysis (PCA) was applied to uncover the underlying structure of the dataset. Since the dataset consisted of both quantitative and qualitative variables, qualitative data were coded as ordinal categories (Hastie et al. 2009) and included in the PCA (Jolliffe 2002). This approach enabled the simultaneous analysis of mixed data types and facilitated a comprehensive evaluation of the dataset.

In the analysis, the Kaiser Normalization was applied along with the Varimax rotation method. Kaiser Normalization standardizes factor loadings, ensuring more stable and comparable results (Kaiser 1958); the Varimax rotation was chosen to improve the interpretability of the principal components. Varimax rotation maximizes the variance explained by each component, thereby better distinguishing the loadings of variables across components (Jackson 2005). This method has contributed to making the factor structure more meaningful, especially in high‐dimensional data (Kaiser 1960).

In selecting the principal components, only those with an eigenvalue greater than 1.00 were considered (Abdi and Williams 2010). This criterion ensured that only components contributing meaningfully to the total variance of the dataset were included in the model, while components with low variance, which are deemed insignificant, were excluded. This method helped reduce noise in the analysis and facilitated obtaining more reliable results.

The rotation process successfully converged after 14 iterations, demonstrating that the model effectively captured the variance in the dataset (Table 4). As a result, PCA both strengthened the relationships between the data and facilitated easier interpretation of the principal components. This methodology provided an effective tool for understanding the complexity of the dataset and assessing the relative importance of the variables.

**TABLE 4:** Principal component analysis (PCA): Eigenvalues and variance explained by the major axes for pomological and morphological traits in the peach accessions.

Trait	Component													
	1	2	3	4	5	6	7	8	9	10	11	12	13	14
Tree growth habit	0.41	0.07	0.33	−0.06	−0.15	−0.33	0.11	0.40	−0.06	−0.13	−0.04	0.11	−0.08	−0.15
Tree growth vigor	0.62	0.22	0.32	0.06	0.27	−0.27	0.07	0.26	0.08	−0.03	0.15	0.29	−0.11	−0.03
Tree height	0.45	0.10	0.13	0.27	0.13	−0.15	0.48	0.39	0.00	0.07	0.15	0.07	−0.25	−0.05
Trunk type	0.00	0.01	−0.21	0.39	0.20	0.34	−0.35	−0.21	−0.08	0.03	0.22	0.38	0.15	−0.02
Canopy density	0.52	0.18	0.29	0.29	0.20	−0.30	0.03	0.28	0.09	−0.18	0.24	0.04	−0.28	0.02
Branching	0.47	0.17	0.30	0.03	0.19	−0.32	0.13	0.24	0.12	0.05	0.19	0.34	0.03	0.02
Flowering date	−0.12	0.06	0.02	0.16	0.13	0.09	0.05	−0.06	**0.85**	−0.03	−0.04	0.04	0.16	−0.06
Flower density	0.17	0.02	**0.84**	−0.09	−0.05	0.02	−0.10	0.03	0.04	0.12	0.08	−0.02	0.02	0.09
Peduncle length	0.13	−0.03	0.12	−0.16	0.34	0.35	0.35	−0.21	0.28	−0.18	0.08	0.10	−0.06	0.34
Peduncle width	0.12	−0.01	−0.02	0.05	−0.13	−0.04	0.11	0.00	−0.05	−0.02	−0.07	**0.84**	0.06	0.07
Petal shape	0.36	0.02	0.02	0.32	−0.23	0.05	0.23	−0.28	−0.29	0.20	0.15	0.30	−0.25	0.19
Petal length	**0.75**	0.15	0.01	0.04	−0.09	0.10	−0.01	0.37	−0.07	0.18	0.20	0.06	−0.08	0.16
Petal width	0.27	0.03	0.01	−0.15	−0.07	0.05	−0.09	**0.84**	0.09	0.09	0.04	0.04	0.15	0.05
Hypanthium length	0.46	0.01	−0.31	−0.10	0.55	0.07	−0.06	0.05	0.09	0.18	0.08	0.03	−0.22	−0.18
Hypanthium diameter	0.31	−0.01	−0.34	0.11	−0.03	0.25	0.16	0.55	0.00	−0.12	0.31	−0.31	−0.14	−0.04
Sepal external color	0.02	0.19	0.04	0.03	−0.48	0.00	−0.15	−0.26	−0.20	−0.03	0.38	−0.20	−0.42	0.07
Sepal length	−0.10	0.04	0.01	0.22	0.17	0.62	−0.04	0.22	0.10	−0.17	0.16	0.18	0.28	0.21
Sepal width	0.01	−0.06	0.11	0.12	0.02	**0.76**	0.05	0.01	−0.16	0.15	−0.09	−0.10	−0.13	0.00
Number of stamens	0.06	0.08	−0.03	0.11	0.12	−0.02	0.12	0.04	0.05	0.16	0.18	0.02	**0.76**	−0.04
Length of the stamen in relation to Carpel	0.03	0.02	0.09	0.05	**0.75**	0.13	0.19	−0.12	0.08	−0.03	−0.05	−0.22	0.17	−0.17
Leaf length	**0.87**	0.01	−0.08	−0.07	0.13	0.04	0.00	0.16	−0.02	0.13	0.18	0.10	−0.11	−0.01
Leaf width	0.19	0.17	0.00	0.08	−0.07	0.02	0.06	0.01	−0.20	**0.82**	0.06	0.04	0.24	−0.03
Petiole length	0.56	−0.08	0.01	−0.21	0.04	−0.17	0.22	−0.09	0.08	0.41	0.19	0.24	0.15	0.23
Petiole diameter	0.53	−0.11	0.03	−0.14	0.21	0.16	0.16	0.14	−0.01	0.49	0.10	−0.11	−0.27	0.10
Leaf shape	0.04	0.18	0.11	**−0.80**	0.09	−0.07	0.13	0.07	−0.24	0.20	0.07	0.04	0.00	0.12
Leaf margin form	0.06	0.07	0.15	−0.05	−0.18	0.09	0.00	0.05	−0.10	0.02	−0.01	0.03	−0.04	**0.83**
Leaf serration shape	−0.02	0.18	−0.15	−0.22	−0.16	0.13	**0.74**	0.13	0.15	0.07	−0.02	−0.10	0.32	0.08
Leaf color	0.07	−0.13	−0.03	−0.03	−0.26	0.05	**−0.77**	0.14	0.06	−0.06	0.07	−0.15	−0.03	0.04
Leaf apex shape	−0.33	−0.19	0.20	**0.74**	0.03	0.16	0.02	0.00	−0.13	0.26	0.01	0.03	0.19	−0.08
Ripening date	0.05	0.03	−0.05	−0.04	−0.02	−0.21	0.01	0.14	**0.84**	−0.10	0.09	−0.06	−0.08	−0.01
Fruit yield	0.21	−0.18	**0.76**	0.21	0.05	0.15	0.10	−0.07	−0.09	−0.10	−0.06	−0.01	−0.09	0.10
Fruit length	**0.93**	0.11	0.08	−0.03	−0.02	0.02	−0.04	0.06	−0.02	0.15	0.05	0.11	0.08	0.06
Fruit width	**0.70**	0.63	0.07	−0.13	−0.09	0.06	0.13	−0.11	0.02	0.01	0.02	0.05	−0.01	−0.10
Fruit diameter	**0.72**	0.60	0.09	−0.07	−0.09	0.08	0.03	−0.09	−0.08	−0.16	0.00	0.00	0.04	−0.10
Fruit weight	**0.83**	0.42	0.18	−0.07	−0.09	−0.02	0.05	0.08	−0.02	0.02	−0.04	0.02	0.05	0.04
Fruit skin blush color	0.50	−0.11	−0.22	−0.09	−0.12	−0.02	−0.01	0.12	−0.18	0.14	0.62	0.09	0.10	−0.02
Fruit flesh thickness	0.54	0.09	0.39	−0.05	−0.37	0.45	0.02	0.02	−0.02	−0.16	0.02	0.13	−0.13	−0.10
Fruit flesh color	0.07	−0.13	0.12	0.02	0.00	−0.01	−0.04	0.07	0.13	0.04	**0.86**	−0.08	0.13	0.01
Fruit flesh percentage	**0.85**	−0.21	0.18	0.04	0.09	−0.18	−0.15	−0.04	0.07	0.03	−0.06	−0.14	0.06	0.19
Depth of fruit stalk cavity	**0.67**	0.25	0.14	0.14	0.00	0.11	−0.05	0.15	−0.15	0.03	−0.16	−0.05	0.12	−0.36
Fruit stalk attachment diameter	0.26	0.24	0.13	−0.10	0.13	0.21	−0.23	0.08	0.18	0.35	−0.13	0.48	−0.32	−0.09
Stone length	0.42	0.26	0.19	0.54	0.12	0.10	0.05	−0.06	0.06	0.07	0.04	0.11	0.02	0.34
Stone width	0.30	**0.78**	−0.05	0.02	0.11	−0.13	0.05	0.05	0.12	0.22	−0.26	−0.06	0.00	0.15
Stone diameter	0.20	**0.84**	−0.11	−0.05	0.17	−0.15	0.00	0.04	0.13	0.07	−0.06	−0.11	0.08	0.20
Stone weight	0.00	**0.85**	−0.03	−0.12	−0.18	0.15	0.24	0.07	−0.11	−0.04	0.05	0.22	−0.03	−0.17
Total soluble solids	−0.17	0.34	−0.09	0.03	0.48	−0.19	−0.33	−0.13	−0.08	−0.32	−0.05	0.18	0.03	0.34
Eigenvalue	10.79	3.54	2.99	2.88	2.62	2.21	2.07	1.88	1.77	1.55	1.50	1.32	1.24	1.10
Component degree of significance	**	**	*	*	*	*	*	*	*	*	*	*	*	*
Variance (%)	23.45	7.71	6.49	6.27	5.70	4.80	4.50	4.08	3.85	3.36	3.27	2.87	2.70	2.39
∑ variance (%)	23.45	31.16	37.65	43.92	49.61	54.41	58.92	63.00	66.85	70.21	73.48	76.34	79.04	81.43

*Note:* Bold values indicate the characteristics that most influence each PC (Eigenvalues ≥ 0.67).

* *p* < 0.05.

** *p* < 0.01.

In this study, the first 14 principal components accounted for 81.43% of the total variation in the dataset. The degree of significance for the components was as follows: PC1 and PC2 were significant at *p* < 0.05, while PC3 was significant at *p* < 0.01.

PC1 accounted for 23.45% of the total observed variation and was predominantly shaped by several key variables, including fruit length (0.93), leaf length (0.87), fruit flesh percentage (0.85), fruit weight (0.83), petal length (0.75), fruit diameter (0.72), fruit width (0.70), and the depth of the fruit stalk cavity (0.67). The high loading values of these traits indicate their substantial influence on this principal component, underscoring the critical role of fruit and flower size, weight, and structural features in driving phenotypic variability among the accessions. Notably, attributes such as larger fruit size and increased weight are considered economically advantageous, both in fresh market sales and fruit processing industries. These features are frequently prioritized in selection programs aimed at identifying superior genotypes (Mars and Marrakchi 1999; Shahkoomahally et al. 2021). Therefore, the findings suggest that traits related to fruit size, leaf dimensions, and flesh proportion are among the most influential factors differentiating the accessions, which is consistent with earlier studies linking these characteristics to overall plant vigor and productivity (Caliskan and Bayazit 2013; Durgac et al. 2008; Nikolić et al. 2010).

PC2, which explained 7.71% of the total variance, was mainly characterized by seed‐related traits, specifically stone weight (0.85), stone diameter (0.84), and stone width (0.78). These results highlight the distinct contribution of seed morphology in shaping the second principal component and suggest that these traits may have implications for reproductive strategies and overall plant performance. Interestingly, the interpretation of PC2 varies across previous studies. While Nikolić et al. (2010) associated it with total soluble solids, Bakić et al. (2017) linked it to floral morphology, and Drogoudi et al. (2023) identified relationships with fruit size. Such inconsistencies underscore the influence of dataset composition and environmental conditions, as well as the specific traits included in each study, contributing to divergent interpretations across the literature.

PC3 accounted for 6.49% of the total variation and was primarily influenced by flower density (0.84) and fruit yield (0.76). This component reflects the importance of floral attributes and fruit productivity in explaining accession performance. The strong association between flower density and yield suggests that reproductive architecture plays a pivotal role in determining the productive potential of genotypes. However, the trait associations identified in PC3 also vary in the literature. For instance, Nikolić et al. (2010) related this component to yield and fruit shape, Bakić et al. (2017) emphasized seed weight and fruit coloration, while Drogoudi et al. (2023) linked it to anthocyanin content. These variations further illustrate the context‐dependent nature of principal component interpretations and point to the uniqueness of the current dataset in comparison with earlier research.

The differences in our findings for PC2 and PC3 compared to those of previous researchers can be attributed to ecological factors as well as the number and diversity of the accessions used. Different ecological conditions can lead to variations in plant morphology and genetic makeup, affecting the expression of such traits. Additionally, the varying number and genetic diversity of the accessions used can influence the results of the analysis, leading to different associations and findings for the same components.

Furthermore, the first three principal components together accounted for 37.65% of the total variation, highlighting their significant role in capturing the underlying patterns of the dataset.

Overall, the first three components capture a substantial portion of the variation, reflecting the complex interplay between fruit, seed, and floral characteristics. This analysis provides valuable insights into the primary factors that drive variability in the studied accessions and can guide future breeding or cultivation strategies focused on optimizing these traits.

The PCA biplot serves as a powerful visualization tool for simplifying the interpretation of complex, high‐dimensional datasets. By simultaneously plotting both the samples (observations) and the quantitative variables on the same two‐dimensional plane, it facilitates a comprehensive understanding of the underlying data structure. This approach reduces dimensionality by identifying the principal components that capture the maximum variance in the dataset. In the biplot, vectors (arrows) indicate the influence and orientation of each variable, whereas the proximity between points reflects the degree of similarity among samples. As such, this graphical representation is especially advantageous for detecting patterns, clusters, and relationships within large and intricate datasets (Johnson and Wichern 2014).

Quantitative data are primarily employed in biplot analyses due to their inherent linearity and scalability. PCA relies on linear transformations to account for the variance within a dataset, making it particularly well‐suited to continuous and numerical variables (Gabriel 1971). On the other hand, categorical or qualitative variables—being inherently non‐numeric and lacking a natural order—do not align with the assumptions of linear modeling. Attempts to numerically encode such variables can lead to distorted or oversimplified interpretations, as their relationships are often nonlinear in nature. Consequently, the use of quantitative data in biplots ensures greater accuracy, interpretability, and methodological consistency in multivariate analyses (Wold et al. 1987).

**FIGURE 4:**
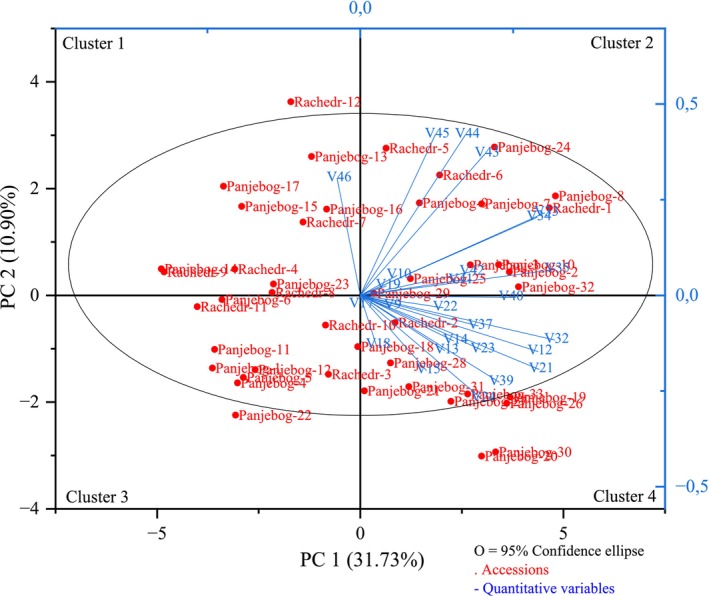
PCA biplot (PC1 vs. PC2) illustrating the distribution of peach accessions and trait vectors based on pomological and morphological characteristics (for abbreviations, see Table 1).

Figure 4 presents the biplot illustrating the distribution of the analyzed peach accessions and the associated quantitative traits based on the first two principal components (PC1 and PC2), derived from morphological and pomological data. Collectively, PC1 and PC2 explain 42.63% of the total observed variance, with PC1 contributing 31.73% and PC2 accounting for 10.90% of the variability.

The biplot analysis revealed a distribution of the accessions and associated quantitative traits across four distinct clusters. Cluster 1 comprised the accessions “Panjebog‐13,” “Panjebog‐14,” “Panjebog‐15,” “Panjebog‐16,” “Panjebog‐17,” “Panjebog‐23,” “Rachedr‐4,” “Rachedr‐7,” “Rachedr‐8,” “Rachedr‐9,” and “Rachedr‐12,” which were closely associated with the variable total soluble solids. This grouping suggests a shared phenotypic profile among these accessions in terms of soluble solids concentration, potentially reflecting similarities in fruit sweetness or quality attributes. Such traits are of considerable interest for selection in breeding programs targeting enhanced flavor or sugar accumulation.

Cluster 2 included “Panjebog‐2,” “Panjebog‐3,” “Panjebog‐7,” “Panjebog‐8,” “Panjebog‐9,” “Panjebog‐10,” “Panjebog‐24,” “Panjebog‐25,” “Panjebog‐29,” “Panjebog‐32,” “Rachedr‐1,” “Rachedr‐5,” and “Rachedr‐6.” These accessions were associated with a range of fruit and seed‐related traits, including peduncle width, number of stamens, fruit width, fruit diameter, fruit weight, fruit stalk attachment diameter, and stone dimensions (length, width, diameter, and weight). Their co‐localization in the biplot indicates convergence in morphological and pomological traits linked to fruit size and stone characteristics—key parameters influencing marketability and breeding potential.

Cluster 3 was formed by the accessions “Panjebog‐1,” “Panjebog‐4,” “Panjebog‐5,” “Panjebog‐6,” “Panjebog‐11,” “Panjebog‐12,” “Panjebog‐18,” “Panjebog‐22,” “Rachedr‐3,” “Rachedr‐10,” and “Rachedr‐11,” all of which were grouped with the variable sepal length. This association implies a degree of uniformity in floral structure, particularly in sepal morphology, among these accessions. As floral traits are often linked to reproductive success and hybridization potential, this cluster may offer insights into the genetic basis of flowering characteristics.

Cluster 4 consisted of “Panjebog‐19,” “Panjebog‐20,” “Panjebog‐21,” “Panjebog‐26,” “Panjebog‐27,” “Panjebog‐28,” “Panjebog‐30,” “Panjebog‐31,” “Panjebog‐33,” and “Rachedr‐2.” These accessions were characterized by strong associations with variables such as peduncle length, petal dimensions, hypanthium size, sepal width, leaf metrics (length, width, petiole length and diameter), fruit length, fruit flesh thickness, flesh percentage, and the depth of the fruit stalk cavity. The traits represented in this cluster reflect an integration of vegetative and reproductive features, underscoring their relevance in determining overall fruit morphology, developmental potential, and adaptation strategies. Such accessions may hold promise for improving both yield and morphological quality in future breeding efforts.

Each cluster represents a natural aggregation of accessions exhibiting similar pomological, morphological, and physiological traits. The patterns observed in the biplot provide critical insights for identifying promising accessions for breeding purposes, elucidating trait interrelationships, and selecting accessions with favorable characteristics.

Notably, the accessions “Rachedr‐12,” “Panjebog‐19,” “Panjebog‐20,” “Panjebog‐22,” “Panjebog‐26,” and “Panjebog‐30” lie outside the 95% confidence ellipse. This ellipse is a statistical boundary that encompasses 95% of the data points in a two‐dimensional space, serving as an indicator of data dispersion and variability (Johnson and Wichern 2018). Accessions falling beyond this boundary are considered outliers, potentially due to unique or extreme trait expressions. These distinct genotypes warrant further investigation to determine the underlying biological or environmental factors contributing to their divergence from the main population structure (Hoover 1984; Mardia et al. 2024).

Importantly, no previous studies were found that applied biplot analysis exclusively using quantitative data in a comparable context. Therefore, the results of this study have been examined in detail and discussed independently to highlight their scientific relevance. This methodological choice offers significant advantages, particularly in terms of enhancing interpretability and analytical precision. By focusing solely on quantitative variables, the analysis allows for direct assessment of the key traits contributing to variation among accessions and facilitates a clearer understanding of trait interactions.

The use of the biplot visualization has proven especially effective in revealing the structural organization of the dataset, clarifying the relationships between variables and accessions. Furthermore, the application of this method with purely quantitative data addresses a gap in the existing literature and strengthens the study's contribution to the field. The in‐depth and compartmentalized discussion of the results enhances the interpretive value of the findings and provides a solid foundation for future research utilizing similar multivariate approaches. Overall, the structured and detailed analysis presented in this study introduces novel perspectives and practical implications for both academic inquiry and applied breeding programs.

### 3.5 Heat Map Analysis (HMA)

The heat map analysis (HMA) based on Ward's method and Euclidean distance coefficients is a widely used analytical technique for clustering data and visualizing similarities. In this analysis, quantitative data are used, and the similarities or differences between data points are calculated using Euclidean distance. Euclidean distance represents the linear distance between two points and is one of the most accurate methods for reflecting relationships between data (Everitt et al. 2011).

Based on the obtained distance matrix, clusters are formed using Ward's method. Ward's method minimizes the variance between groups, ensuring the formation of homogeneous subgroups. This method is particularly preferred in classification analyses where inter‐cluster differences need to be minimized (Kaufman and Rousseeuw 2009; Ward 1963).

The heat map is used to visualize the results of this analysis and helps in understanding the relationships between both observations (samples) and quantitative variables. On the heat map, color intensity represents the magnitude of distance or similarity between the data points. For example, similar samples are represented with closer color shades, while different samples are shown with more distinct color tones (Wilkinson and Friendly 2009).

This method is especially preferred for analyzing large and complex datasets because it visually reveals the clustering structure of the data quickly and effectively. Thus, similarities, differences, and groupings among the samples can be identified.

**FIGURE 5:**
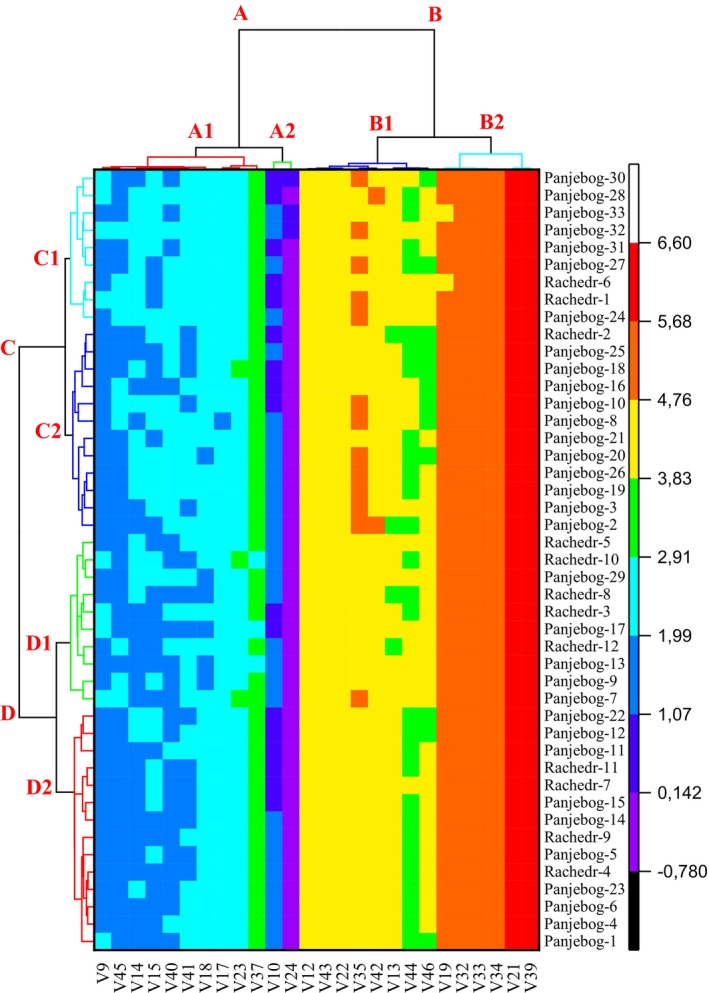
Heat map visualization of hierarchical clustering based on pomological and morphological traits in the peach accessions (for abbreviations, see Table 1).

In this study, the heat map created using Ward's method and Euclidean distance coefficients visualized the similarities and differences among the analyzed samples. The analysis process was performed using quantitative data, and the results reveal how the data are clustered into groups and which samples are more closely related to each other (Figure 5).

The variables were initially divided into two main groups, A and B, which were subsequently subdivided into two subgroups each (A1, A2, B1, and B2). Subgroup A1 included morphological traits such as peduncle length, stone weight, hypanthium length, hypanthium diameter, depth of fruit stalk cavity, fruit stalk attachment diameter, sepal width, sepal length, petiole length, and fruit flesh thickness, which are primarily associated with the structural dimensions of the fruit and related plant parts. In contrast, subgroup A2 comprised peduncle width and petiole diameter, two variables reflecting specific structural measurements that appear less interdependent with the broader set of traits in subgroup A1. Group B included variables associated with fruit size. Subgroup B1 contained petal length, stone width, leaf width, fruit weight, stone length, petal width, stone diameter, and total soluble solids, all of which are strongly linked to fruit size, weight, and overall quality. Subgroup B2 included the number of stamens, fruit length, fruit width, fruit diameter, leaf length, and fruit flesh percentage, variables that reflect compositional and dimensional traits influencing fruit yield and structure.

The accessions were similarly divided into two main groups, C and D, which were further split into two subgroups each (C1, C2, D1, and D2). Subgroup C1 comprised “Panjebog‐30,” “Panjebog‐28,” “Panjebog‐33,” “Panjebog‐32,” “Panjebog‐31,” “Panjebog‐27,” “Rachedr‐6,” “Rachedr‐1,” and “Panjebog‐24,” accessions that clustered together likely due to shared morphological similarities. Subgroup C2 included accessions “Rachedr‐2,” “Panjebog‐25,” “Panjebog‐18,” “Panjebog‐16,” “Panjebog‐10,” “Panjebog‐8,” “Panjebog‐21,” “Panjebog‐20,” “Panjebog‐26,” “Panjebog‐19,” “Panjebog‐3,” and “Panjebog‐2,” reflecting a more heterogeneous group with intermediate traits. In group D, subgroup D1 consisted of “Rachedr‐5,” “Rachedr‐10,” “Panjebog‐29,” “Rachedr‐8,” “Rachedr‐3,” “Panjebog‐17,” “Rachedr‐12,” “Panjebog‐13,” “Panjebog‐9,” and “Panjebog‐7,” a mixture of Panjebog and Rachedr accessions that share overlapping characteristics, potentially indicating functional or developmental similarities. Subgroup D2, which was the largest and most diverse cluster, included “Panjebog‐22,” “Panjebog‐12,” “Panjebog‐11,” “Rachedr‐11,” “Rachedr‐7,” “Panjebog‐15,” “Panjebog‐14,” “Rachedr‐9,” “Panjebog‐5,” “Rachedr‐4,” “Panjebog‐23,” “Panjebog‐6,” “Panjebog‐4,” and “Panjebog‐1,” representing a broader range of variation, suggesting a group with potentially unique or distinguishing traits.

Accessions in the C1 subgroup were influenced by structural traits such as peduncle length, stone weight, hypanthium length, hypanthium diameter, and fruit flesh thickness. These variables are generally associated with fruit size and weight and played a key role in the clustering of these accessions. In contrast, accessions in the C2 subgroup were predominantly influenced by more specific structural variables, such as peduncle width and petiole diameter. These traits contributed to the differentiation of this group from the others. Additionally, fruit length, fruit width, and leaf size were also prominent in this group. Accessions in the D1 group were more influenced by larger fruit characteristics, such as fruit weight and stone length. This group predominantly included accessions with larger and heavier fruit traits. Accessions in the D2 subgroup, which were the most heterogeneous, were influenced by a more complex set of traits, including fruit flesh thickness, stone diameter, and sepal length. This group stood out for its significant phenotypic diversity.

Thus, both the variables and the accessions were examined individually and concerning each other through a comparative analysis. This approach not only clarified the specific characteristics of each variable and accession but also facilitated a deeper understanding of the interactions between different traits. The detailed analysis conducted on both individual and group levels allowed for a more comprehensive interpretation of the data. Furthermore, this method visually represented how the pomological and morphological characteristics of each accession either align or differ, enhancing the accuracy and reliability of the findings. In this way, it became possible to better comprehend the overall structure and dynamics within the dataset.

The strength of heat map analysis was highlighted as a robust tool for visualizing complex datasets, enabling clear identification of relationships both within and between clusters. By grouping accessions and variables based on shared traits, the analysis provided a comprehensive understanding of the dataset's structure. The findings not only enhanced the clarity of the relationships among accessions but also offered a solid foundation for future studies. This approach could guide the identification of accessions with desirable traits for breeding programs or further genetic analyses, making the analysis an invaluable resource for understanding phenotypic variation and supporting future research directions.

## 4 Conclusions

This study comprehensively evaluated pomological, morphological, and physiological traits in a diverse set of peach accessions using multivariate analysis. The results revealed substantial phenotypic variation among accessions, particularly in fruit size, flesh percentage, leaf dimensions, and floral attributes. PCA and biplot analyses identified the most influential variables contributing to this variation and enabled the classification of accessions into distinct groups based on shared characteristics.

Among the studied accessions, the top 15 accessions with the highest scores based on individual quantitative traits were identified as “Panjebog‐19,” “Panjebog‐26,” “Rachedr‐1,” “Panjebog‐20,” “Panjebog‐2,” “Panjebog‐8,” “Panjebog‐24,” “Panjebog‐30,” “Panjebog‐32,” “Panjebog‐10,” “Panjebog‐3,” “Panjebog‐7,” “Panjebog‐33,” “Rachedr‐5,” and “Panjebog‐27.” Notably, “Panjebog‐19” and “Panjebog‐20” were located outside the 95% confidence ellipse, suggesting that these accessions possess distinctive trait combinations. Their outstanding performance indicates strong potential as promising candidates for future breeding programs focused on developing superior cultivars with enhanced yield and fruit quality.

The strong correlations identified between fruit traits (such as length, weight, and flesh percentage), floral dimensions (including petal and sepal length), and leaf morphology highlight the interconnected nature of vegetative and reproductive development in peach. These associations offer valuable insight into trait integration and their utility in genotype selection.

As previous studies employing biplot analysis exclusively with quantitative data in peach are limited, this work contributes significantly to the existing literature by establishing a novel analytical approach. Future research should investigate the genetic determinants of the observed phenotypic diversity and evaluate trait stability across varying environmental conditions, thereby supporting marker‐assisted selection and targeted breeding strategies.

## Author Contributions

**Ali Khadivi:** conceptualization (equal), formal analysis (equal), investigation (equal), methodology (equal), project administration (equal), writing – review and editing (equal). **Farhad Mirheidari:** investigation (equal). **Abdolvahid Saeidifar:** investigation (equal). **Younes Moradi:** investigation (equal). **Yazgan Tunç:** formal analysis (equal), methodology (equal), writing – original draft (lead), writing – review and editing (equal). **Daya Shankar Mishra:** writing – original draft (equal), writing – review and editing (equal).

## Disclosure

For this study, we acquired permission to study peach issued by the Agricultural and Natural Resources Ministry of Iran. The either cultivated or wild‐growing plants sampled comply with relevant institutional, national, and international guidelines and domestic legislation of Iran.

## Ethics Statement

The authors has nothing to report.

## Consent

The authors have nothing to report.

## Conflicts of Interest

The authors declare no conflicts of interest.

## Data Availability

The data that support the findings of this study are available from the corresponding author upon reasonable request.
